# Active Acquisition for multimodal neuroimaging

**DOI:** 10.12688/wellcomeopenres.14918.2

**Published:** 2019-09-23

**Authors:** James H. Cole, Romy Lorenz, Fatemeh Geranmayeh, Tobias Wood, Peter Hellyer, Steven Williams, Federico Turkheimer, Rob Leech

**Affiliations:** 1 Department of Neuroimaging, Institute of Psychiatry, Psychology and Neurosciences, King's College London, London, UK; 2 MRC Centre for Cognition and Brain Sciences, University of Cambridge, Cambridge, UK; 3 Max Planck Institute for Human Cognitive and Brain Sciences, Leipzig, Germany; 4 Department of Brain Sciences, Faculty of Medicine, Imperial College London, London, UK

**Keywords:** Neuroimaging, active acquisition, MRI, brain

## Abstract

In many clinical and scientific situations the optimal neuroimaging sequence may not be known prior to scanning and may differ for each individual being scanned, depending on the exact nature and location of abnormalities. Despite this, the standard approach to data acquisition, in such situations, is to specify the sequence of neuroimaging scans prior to data acquisition and to apply the same scans to all individuals. In this paper, we propose and illustrate an alternative approach, in which data would be analysed as it is acquired and used to choose the future scanning sequence: Active Acquisition. We propose three Active Acquisition scenarios based around multiple MRI modalities. In Scenario 1, we propose a simple use of near-real time analysis to decide whether to acquire more or higher resolution data, or acquire data with a different field **-**of **-**view. In Scenario 2, we simulate how multimodal MR data could be actively acquired and combined with a decision tree to classify a known outcome variable (in the simple example here, age). In Scenario 3, we simulate using Bayesian optimisation to actively search across multiple MRI modalities to find those which are most abnormal. These simulations suggest that by actively acquiring data, the scanning sequence can be adapted to each individual. We also consider the many outstanding practical and technical challenges involving normative data acquisition, MR physics, statistical modelling and clinical relevance. Despite these, we argue that Active Acquisition allows for potentially far more powerful, sensitive or rapid data acquisition, and may open up different perspectives on individual differences, clinical conditions, and biomarker discovery.

## Introduction

Neuroimaging involves trade-offs; whether for clinical diagnosis, patient stratification, or biomarker discovery. For example, with a typical MRI scan, there are substantial practical constraints (money, patient comfort and compliance, radiological reporting) which means decisions have to be taken as to what kind of scan to perform, where in the brain scan, and the scan resolution. The standard approach is to make these decisions before scanning commences, acquiring the data then analysing it. However, the optimal resolution/type of scan will depend on what is being investigated, and the type and location of the pathology or abnormalities, and may not be known *a priori*.

Here, we propose an alternative approach using active learning for real-time optimisation of neuroimaging data acquisition; providing illustrative examples. Broadly, in our approach data acquisition and analysis are not separated; instead data is analysed as it is acquired and used to guide subsequent data acquisition, in a closed-loop. The word game *hangman* is a simple illustration of a form of active learning (as is predictive text messages and search engine auto-completion): a letter is guessed, and whether it is present or not is then evaluated; this information is then used to narrow the search for the next letter. Active learning approaches are potentially far more efficient (in terms of scanner time) than treating acquisition and analysis as separate phases. A non-active learning version of hangman would involve guessing all the letters at the start of the game and then evaluating them all at once without any feedback; in most situations, this would be a highly inefficient strategy.

We have previously demonstrated that active learning can be used to guide the choice of experimental paradigm in functional MRI ( Lorenz *et al*., 2016): with substantial increases in terms of speed, searching over many experimental parameters far quicker than an exhaustive search. This allows for far broader research questions to be asked ( Lorenz *et al*., 2018). Active learning also has another important feature; it involves a prediction and testing cycle, with the learner having to make predictions that are then tested with out-of-sample data. This potentially increases the replicability of analyses and reduces the ability for post-hoc bias ( Lancaster *et al*., 2018; Lorenz *et al*., 2017).

The work presented here investigates the use of sequential decision-making to select the type of scan, using information gained from previous scans to actively seek out brain abnormalities or make diagnostic predictions. This requires data to be collected and analysed in near real-time; however, to illustrate the potential power of this approach our demonstrations use previously collected data, by simulating the real-time analysis aspect.

Video 1 presents a video overview of active acquisition: (i) scan parameters are chosen (e.g., modality or acquisition parameters such as resolution, repetition time or echo time); (ii) the scan is acquired; (iii) pre-processed; and, (iv) acquired data is compared to an existing normative dataset. The loop then continues with the information in (iv) used to optimise the next scan (or decide whether sufficient data have been collected to stop scanning). We explore using Active Acquisition in three different scenarios with T1-weighted MR images:

1) Finding a localised structural anomaly (e.g., locating a focal lesion).
2) Choosing the optimal scanning modality to actively detect abnormalities.
3) Actively choosing the type of scan to characterize an aspect of the individual being scanned (e.g., age).

Video 1. General illustrative video of one active acquisition approach for structural neuroimaging. General illustrative video of one active acquisition approach for structural neuroimaging. Click here for additional data file. Copyright: © 2019 Cole JH et al. 2019

## Methods

### Scenario 1: Changing structural scan resolution to detect stroke pathology

Our rationale is to start at a low image resolution for a (very rapid) whole brain scan, before acquiring higher resolution scans if the brain appears to be abnormal. This way, it is possible to efficiently image a focal pathology, such as a lesion or tumour, and to rapidly estimate its spatial location and establish whether more data needs to be acquired, potentially with a restricted field of view focused on the site of the abnormality.

***Choice of scan parameters.*** For our illustrative simulation, we used structural scans collected offline (Dataset 1 ( Leech & Cole, 2018)). Further details on the participants are included in the Data Acquisition section. In practice they would be acquired and analysed online. Practical challenges and limitations to acquiring these data, as well as consider possible methods to mitigate these challenges, as outlined in the Discussion section.

At each iteration (in terms of increasing resolution), the scan is divided into three equally sized volumes, along the z-dimension. The ‘outlier distance’ (defined below) is then quantified for each third by reference to the distribution in an independent normative sample (in this example, the n=7 healthy controls). The volume with the highest outlier distance is then selected and the next scan “acquired”; covering the same section of the volume, but with the resolution doubled. The process was repeated three times until the maximum resolution of 1mm ^3^ voxel was achieved. The choice of resolution and number of sub-divisions (and other scanner parameters) presented in this scenario is relatively arbitrary. Future work will need to establish the optimal approach for a given clinical or scientific question. There will always be a trade-off between multiple comparisons and precision when assessing; here, because the aim was to illustrate the logic and operation of the approach, we chose a very coarse approach which should be sufficient, given the focal and macroscopic nature of the brain injury (i.e., lesion). In clinical or scientific applications, a more sophisticated approach would probably be required, that chooses the brain region for the outlier detection (and potentially subsequent more targeted acquisition), related to the size and location of the pathology or abnormality, possibly changing orientation, and the image field-of-view in the process. In this vein, different potential decompositions of the multivariable imaging signal could be applied in parallel and evaluated in terms of outlier distance to normative data; subsequently, a decision could then be made (based on e.g., which is most likely an outlier) as to what decomposition to use to guide further data acquisition.

***Outlier distance from normative sample.*** The extent to which a participant’s image was different from the normative sample was quantified, restricted to the resolution and coverage of the specific scan. The median distance between an individual’s scan and each participant in the normative dataset was calculated using the median absolute deviation (in Euclidean distance) of signal intensity averaged across all voxels. This results in a single value of outlier distance. The choice of outlier quantification depends on the type of data being acquired and the question being asked. We opted for the median absolute deviation because it is a simple measure that is relatively robust to violations of normality assumptions. However, we note that many other more sensitive outlier measures could also be used (e.g., measures taking into account covariance across voxels, ( Fritsch *et al*., 2012)).

### Scenario 2: Active multimodal stratification of individual differences

In this scenario, Active Acquisition is used to choose the modality of the scan to achieve a given goal. The rationale is that the optimal scanning modality for assessing an individual, for example to quantify their relationship to a normative sample, will vary for different individuals; when performing a battery of scans, each individual may have a different set of scans and a different acquisition order.

In Scenario 2, we use multimodal imaging to quantify individual variability. This type of analysis could be relevant when classifying or stratifying individuals into scientifically or clinically relevant groups. To illustrate this, we use the Cam-CAN dataset ( Shafto *et al*., 2014) and with the task of predicting chronological age from neuroimaging data. Predicting age is a useful example case for active multimodal imaging because there are large datasets available, there is little ambiguity about label validity (unlike many clinical descriptions), age is associated with large-scale neural changes (e.g., ( Good *et al*., 2001) and “brain-predicted age” has been shown to relate to many other health related biomarkers (e.g., Cole *et al*., 2018a). Cam-CAN is a particularly useful dataset to assess this source of individual variability since the age distribution of the participants is approximately equally balanced across seven decades from 20s to 80s. For details on the modalities please see the Data Acquisition section.

To instigate Active Acquisition in this case, we simulate the active learning process by fitting a decision tree regression model to the six modalities of Cam-CAN; predictions of chronological age were the outcome measure. This is because: a low-depth decision tree would not include all modalities, just those important for predicting age; making the decision sequentially (i.e., modality by modality) rather than simultaneously, thus it is well-suited for Active Acquisition, and finally; allows different individuals to have different scans and different orders of scans.

A holdout dataset was created with 20% of the individuals, selected randomly (the data partition was performed once rather than pooling across multiple, randomly generated partitions). A decision tree was fit to the remaining 80% of individuals’ six imaging modalities as the predictor variables and their ages in years as the outcome variable. The model hyper-parameters (tree depth, number of leaves, etc.) were estimated with Bayesian optimisation ( Leech & Cole, 2018). Subsequently, the decision tree was evaluated with the holdout participants. The application of the decision tree (the sequential decision process) to each individual in the holdout group, could be performed in real-time to new participants in exactly the same way. For comparison, we also fit a standard support vector regression, with hyper-parameters also optimised with Bayesian optimisation, to the same data (see Matlab code, ( Leech & Cole, 2018)) which used all data modalities simultaneously.

### Scenario 3: Active discovery of individual differences with multi-modal imaging

Whereas Scenario 2 focuses on quantifying how an individual varies along some dimension (e.g., age), in Scenario 3, we attempt to actively learn in which modality an individual is most likely to be an outlier (i.e., is a given individual more likely to be an outlier from the norm when using phenotypes derived from: T1-weighted MRI, diffusion-MRI or functional MRI?). we attempt to actively learn which modality an individual is most likely to be an outlier in. This could be useful for efficiently finding pathology in an individual or for discovering biomarkers; particularly, when there are a large number of possible modalities to choose from and a limited amount of scanning time/participant tolerance of scanning ( Leech & Cole, 2018).

To illustrate Scenario 3, we again used the Cam-CAN dataset, as per Scenario 2. In addition, we included a Bayesian optimisation algorithm ( Shahriari *et al*., 2016) to actively learn which modality is most abnormal (as quantified by the magnitude of outlier measurement). Bayesian optimisation is particularly well suited for this type of problem when the the general objective underlying function is unknown a priori and is costly to evaluate at any given point; Bayesian optimization is also relatively robust to the presence of noise in the data.

For optimisation to work efficiently, the acquisition function needs to take advantage of existing information; in this case the covariance across individuals for different modalities. Therefore, we split the data into two: 80% of the Cam-CAN participants were used to estimate the space (across modalities) for the algorithm to search across. To do this, we calculated a single summary statistic for each individual for each modality. Then, we normalised the data within each modality so that each individual had a z-score for each modality. Subsequently, we performed a factor analysis (using Matlab) on the z-scored summary statistics resulting in a single factor. We then reorganized the modalities for the search space for the Bayesian optimisation in terms of weighting on the principle factor; this process estimates how different modalities will co-vary across individuals (approximately) with each other. For this example, with only six modalities to choose from, we opted for a simple experiment space with modalities given an integer between 1–6, based on the output of the factor analysis and the optimisation algorithm output integers. For more realistic situations with more complicated spaces (e.g., with many modalities organised along multiple dimensions and with more continuous modalities) one could use alternative (e.g., ratio) scales.

Subsequently, we performed Bayesian optimisation using the remaining 20% of participants, allowing the algorithm to pick the modality for a given individual, with the target objective of finding the minimum z-score. Given the relatively small number of available modalities, we allowed the algorithm to randomly choose three modalities (the burn-in phase) to sample first, to fit a Gaussian process regression and then to use the expected-improvement acquisition function to choose the next point to sample. The expectation was that after some initial random exploration, the model should be able to take advantage of the covariance across individuals to estimate the modality with the minimum z-score more frequently than expected by chance. Our choice of using the minmum z-score should be considered as illustrative and relatively arbitrary; we could equally have maximised the z-score or the absolute z-score. The actual target value would be based on the clinical/scientific question.

To assess whether this optimisation approach was performing above chance levels, we compared results for each individual with the correct factor ordering of modality (based on the covariance structure across individuals) with a random ordering of modalities. For each individual the order of the modalities was randomised (i.e., the ordering of the modalities was no longer based on prior information about how individuals co-vary across modalities). For both random and true covariance models, we calculated the proportion of participants where the optimisation algorithm correctly found the minimum z-score modality. This assessment process was repeated 100 times with different random seeds, allowing different burn-in sampling trajectories for each individual for each iteration.

### Data acquisition

In Scenario 1, data were acquired from 13 participants: seven healthy controls with no history of neurological problems (average age= 56, range = 46 to 67, female=4); and, six patients with chronic left-hemisphere middle-cerebral artery focal strokes (average age= 60, range = 47 to 78, female=2, average lesion volume= 10.6 cm ^3^). For each participant, three T1-weighted scans were acquired at different voxel resolutions: 1mm ^3^, 2mm ^3^, 4mm ^3^. As with other data presented here, the patient and control data were not collected in real-time, but is intended to illustrate the general utility of the approach. In this example, we use seven healthy controls as a “normative” sample; this is, obviously, far too small for actual practical uses, but was limited by the data set available (multiple resolutions per individual) but does illustrate the potential of the approach if scaled-up.

For Scenarios 2 and 3, multimodal MRI data from 611 people (age range from 18–88, 312, female) were taken from the Cam-CAN dataset. These data consisted of T1-weighted, T2-weighted, diffusion-weighted MRI, three functional scans (resting-state fMRI, movie-watching fMRI and a blocked sensorimotor task-based fMRI). Imaging acquisition has been presented in detail elsewhere ( Shafto *et al*., 2014). Only Cam-CAN participants with complete data from these six sequences were included (n=611, out of n=653).

### Implementation

All analyses were performed with Matlab Version 2017b. Given the relatively low computational requirements of the analyses reported here, there are no additional minimum system requirements over and above those required to run this version of Matlab. Matlab code and associated data is available at Github ( Leech & Cole, 2018).

*Operation*

### Image pre-processing

*Scenario 1:*

To explore the feasibility of processing brain images in near real-time and to make minimal assumptions about the location or nature of pathology when calculating outlier distance, we used very simple and rapid pre-processing. T1-weighted images were converted from DICOM to NifTI format before being linearly-registered into MNI152 1mm ^3^ space using the very efficient registration tool NiftyReg Version 1.3.9 ( Modat, 2012). The same process was performed for each of the three different image resolutions acquired.

*Scenario 2 & 3:*

***T1-weighted MRI.*** All T1-weighted structural images from all three datasets were processed in the same way as follows. Grey matter (GM), white matter (WM) and cerebrospinal fluid (CSF) volumes were calculated using SPM12 ‘Segment’ (University College London, UK). Voxelwise assessment of changes to brain volume was calculated using the SPM symmetric diffeomorphic registration process ( Ashburner, 2007) to a predefined template used in our previous studies ( Cole *et al*., 2018b).

For the Cam-CAN dataset, the other modalities were (briefly) processed as detailed below. These analyses are merely illustrative of the type of data that could be extracted; they have been simplified from multivariate raw data for each individual into a single summary statistic, chosen for its simplicity rather than because it is optimal for measuring individual variability.

***Diffusion-weighted MRI.*** White-matter microstructure was analysed under the diffusion tensor imaging (DTI) paradigm, using FSL Version 5.010 tract-based spatial statistics ( Smith *et al*., 2006) with DTI-TK ( Zhang *et al*., 2007) software for affine then non-linear tensor-based image registration. Normalised tensor images were used to derive voxelwise measures of fractional anisotropy (FA) and mean diffusivity. Mean values across major WM fibre tracts, taken from the JHU-ICBM tract atlas, were calculated, resulting in an FA average value per individual.

***T2-weighted MRI.*** The same diffeomorphic transformation that was calculated for the grey matter was applied to the T2-weighted scan data to warp each individual’s data into the same T1-weighted template space. Subsequently, the average T2-weighted intensity values from the normalised image was calculated.

***Resting state functional connectivity.*** Measures of ‘within-network’ connectivity were calculated from resting-state fMRI data using FSL ‘Dual Regression’ ( Filippini *et al*., 2009). Prior to the dual regression, the standard FSL ‘MELODIC’ analysis pipeline was applied ( Smith *et al*., 2004): high-pass temporal filtering at 100s, spatial smoothing at 5mm FWHM, global intensity normalisation, motion-correction followed by realigning the data into MNI152 space using linear registration before the data were resampled into 4x4x4mm voxel space. Then the data were cleaned by linearly regressing six motion parameters from each voxel’s time-course, before nuisance WM and CSF time-courses were linearly regressed from each voxel (using average CSF and WM masks from the segmentation). Subsequently, using canonical spatial maps of twenty networks (including both intrinsic connectivity networks and likely noise networks) ( Smith *et al*., 2009), cleaned data underwent a multiple regression to derive voxelwise measures of connectivity for each network for each individual. Finally, to keep this aspect of the approach as simple as possible, we averaged all voxels within the default mode network (DMN) mask; this process resulted in an individualized ‘within-network’ connectivity measure for the DMN. Future work (with any fMRI data) could explore only using short segments of the functional time-series (rather than the whole scan), to allow for faster, repeated measurements.

***Movie-watching functional connectivity.*** This was identical to the analysis of the resting state connectivity, calculating individualised within-DMN functional connectivity while watching the movie.

***Task fMRI.*** The sensorimotor task data were analysed following a standard FSL pipeline: global intensity normalisation, high-pass temporal filtering at 100s, spatial smoothing at 5mm FWHM, motion-correction, registration of the data into MNI152 space using linear registration. Subsequently, a general linear model was applied voxelwise (using the standard FSL approach for dealing with the auto-correlation of residuals ( Smith *et al*., 2004)), with separate explanatory variables modelling auditory and visual blocks convolved with a canonical hemodynamic response function. Subsequently, a contrast of all task conditions versus the implicit baseline was calculated and a higher-level group mixed-effects model was used to calculate increased and decreased BOLD activity with task. This resulted in group task positive and task negative networks which were converted into binary mask defined by voxels that survived cluster correction for multiple comparisons. An individualised task fMRI measure was calculated by taking the average activity within the positive network mask and subtracting the average value from the negative network mask.

## Results

### Scenario 1: Changing structural scan resolution to detect stroke pathology

**Figure 1.**
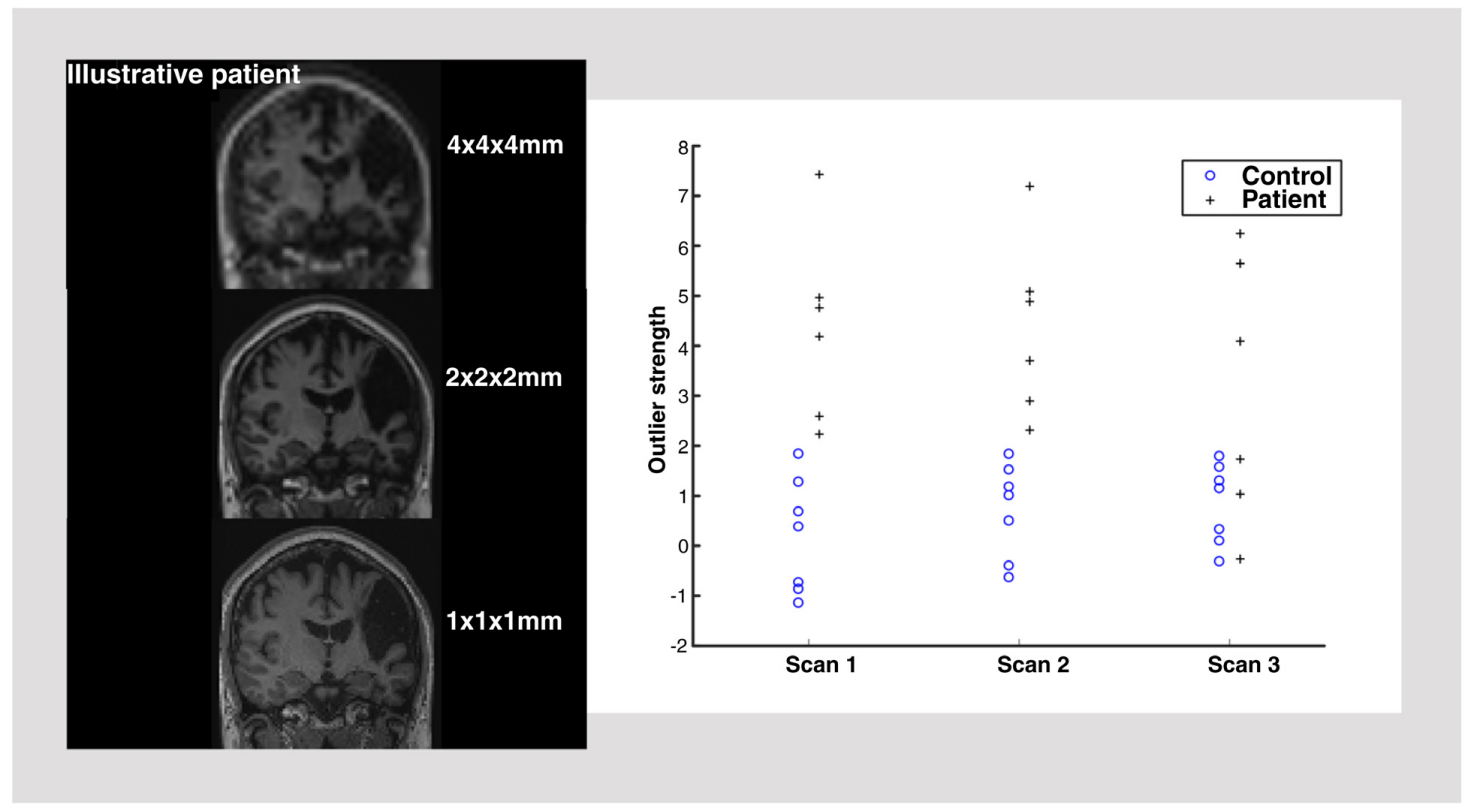
Left, a stroke patient with three different resolution T1-weigthed scans. Left, a stroke patient with three different resolution T1-weigthed scans. Right, outlier distance from control participants, for each participant for the three different scans, and combining all three scans. For each scan, the scan is subdivided into three, and the maximum outlier distance (out of the three subdivisions assessed) from the control data is plotted. This shows a relatively clear difference in outlier distance between patients and controls. For most patients and controls (either far from 0 or close to 0 respectively), there is no need to collect additional higher resolution (slower) scans to differentiate the two groups.

The simplest Active Acquisition model involves starting with a rapid, low resolution structural scan, analysing it and then deciding to whether to acquire further higher-resolution scan(s). Here, we collected lower-resolution (4mm ^3^) structural scans from six patients with focal brain lesions and seven age-matched controls, followed by intermediate-resolution (2mm ^3^) and higher-resolution (1mm ^3^) scans. An illustrative patient at three resolutions is presented in Figure 1 (left). Even with the lowest resolution scan, patients and control participants ( Figure 1 - right) show a large difference in terms of outlier distance. This example, in patients with large focal strokes, illustrates how data simple measures calculated in near real time, and then a decision made as to whether a slower, higher resolution scan is needed or not. As can be seen from the outlier measurements, only a subset of the control participants, close to the boundary with the patients would require slower, additional scans.

**Figure 2.**
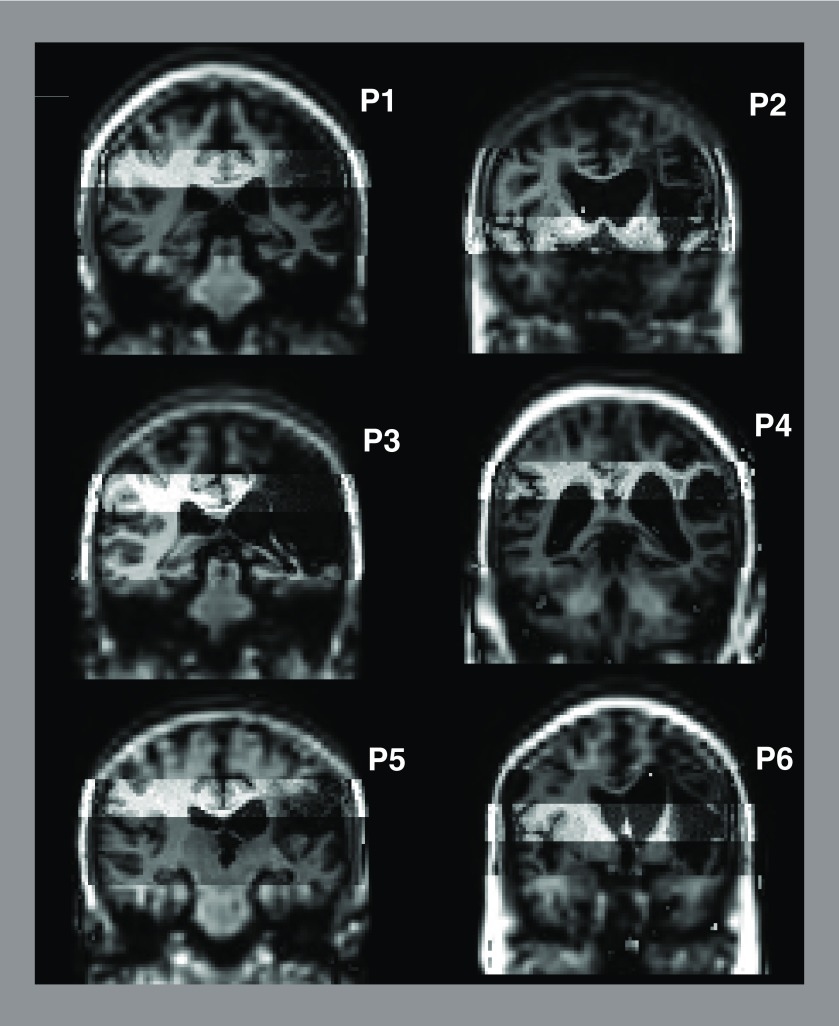
top, composite T1-weigthed coronal slices (one for each of the six patients), comprise out three scans with increasing resolution, each with different coverage of the field-of-view (less field-of-view, higher resolution). The reduced field-of-view is centred on the slice from the previous image that is quantified as most abnormal relative to the norm (i.e., the control group). This demonstrates that the very simple approach to subdividing the brain and quantifying outliers can be used to ‘zoom’ in on areas of pathology that are specific for individual patients.

We also simulated optimising the scan field-of-view in near-real-time. In this case, at each resolution the brain is divided into thirds, and the negative outlier distance calculated for each third. The third that is most strongly classed as an outlier is then retained and subsequent, higher resolution scans, acquired just within that third. The process then repeats ( Figure 2, top). This illustrates how a composite brain image can be built up out of increasing resolution scans. This could trade-off sensitivity for tissue contrast with increasing quantification of brain structure, while limiting scanning time.

### Scenario 2: Active multimodal stratification of individual differences

**Figure 3.**
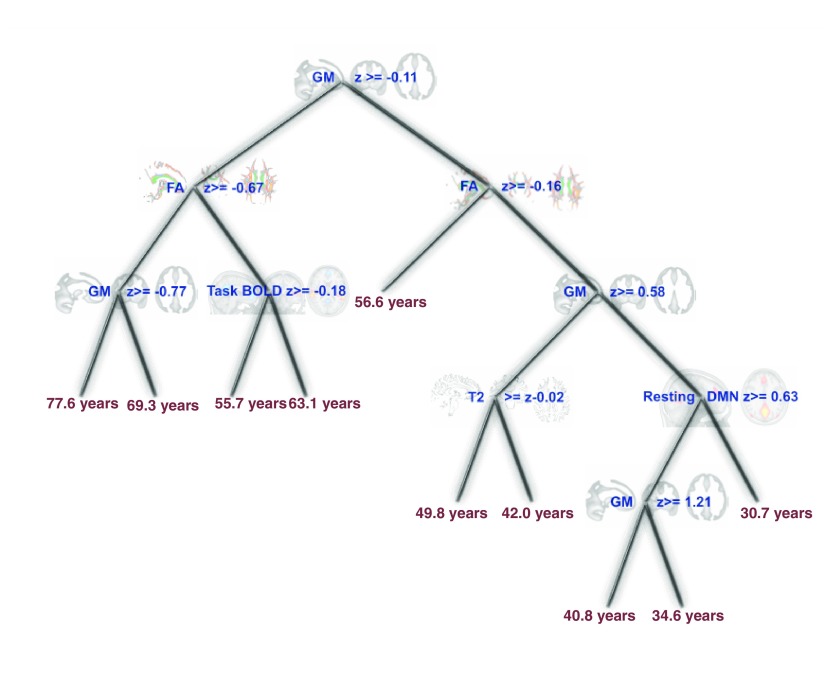
The decision tree regression model calculated on summary statistics for each of six modalities to predict individual age. At each node in the tree the z-scored data for a given individual are used to decide modality to use next or whether to stop at this point. This can happen in near-real time, with different individuals taking different routes through the tree, and with different numbers of scans. The estimated age is then approximated by the age at the leaf nodes.

When fitting the decision tree regression ( Figure 3) to predict chronological age from neuroimaging data, the regression model contained multiple modalities (indicating its utility in a sequential acquisition and analysis procedure). It started with GM volume, consistent with previous data suggesting a strong relationship between GM and age ( Good *et al*., 2001), with lower z-scores indicating older age. Subsequently, average WM FA was chosen, again with lower values relating to older age. Next, the model’s branches become very different, both in terms of modality chosen and number of scans required, depending on the route through the tree. By way of individual examples, if a participant had a GM z-score = -0.8 and FA z-score = -0.7, then they would have a predicted age of 77.6 years (following the left most branches of the decision tree in Figure 3). However, if a participant also has GM z-score = -0.8, but their FA z-score = 0.5, then task BOLD data would be necessary to make a prediction. If that individual’s task BOLD z-score >= -0.18 then their predicted age would be 55.7 years, whereas a task BOLD z-score < -0.18 would give a predicted age of 63.1 years.

We observed that the mean absolute error (MAE) of age prediction is 10.47 years and the median error 8 years. For comparison, the MAE calculated on the same data using a support vector regression approach with all of the data is very similar, was 10.42 years, with a median error of 9.4 years. The predicted age performance is considerably worse than has been reported elsewhere for single modalities from the same dataset e.g., ( Lancaster *et al*., 2018); this is to be expected given that, for illustrative simplicity, we have collapsed large, multivariable datasets into single summary statistics (i.e., a single value for grey matter probability per individualm etcetera). In practice, sequential decision methods incorporating multivariate datasets to utilise the full richness of the underlying data are needed to realise the potential of the approach.

### Scenario 3: Active discovery of individual differences with multi-modal imaging

**Figure 4.**
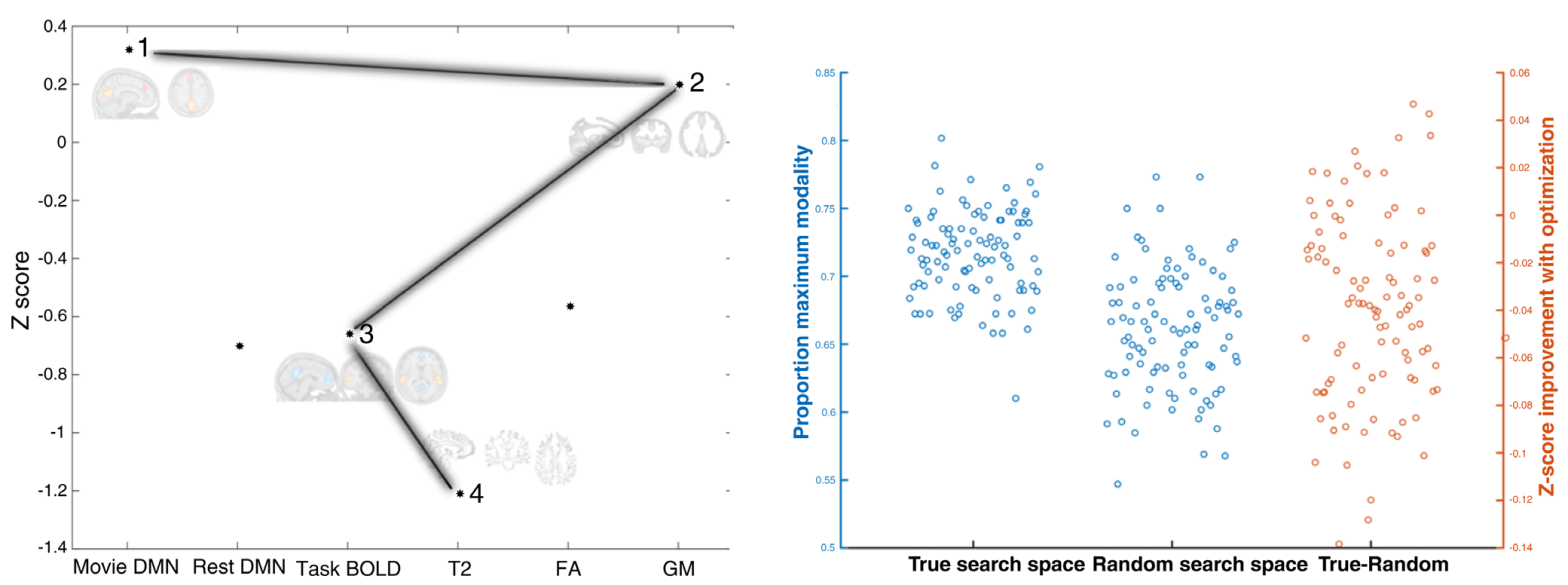
Active discovery of individual differences across modalities, controlled by a closed-loop optimisation algorithm. Left, the trajectory of the algorithm as it traverses the modality space, estimating a model of which modalities a specific individual appears most abnormal in, without exhaustively sampling every point, before guessing which is the most abnormal. Right, proportion of participants in the holdout set where the optimisation algorithm correctly chose the modality most sensitive to abnormalities for both true and random modalities, and the decrease in estimated minimum z-statistic for true versus random organisation of modalities (repeated 100 times with different burn-in random initialisation of the models).

Scenario 3 used a normal distribution (from a normative dataset) to derive individual z-scores for each MRI modality, with the goal of identifying for each individual in which modality they are most outlying (i.e., which modality has the highest absolute z-score for that person). To achieve this we simulated closed-loop Bayesian optimisation to identify the highest z-score from across all modalities for a given individual (from the holdout dataset), as shown in Figure 4. For individual depicted in Figure 4A, the highest z-score was in T2-weighted MRI (z = -1.2), followed by resting-state fMRI, sensorimotor task BOLD, FA, movie BOLD and then GM. This suggests that for this individual, the T2-weighted is likely to be most valuable in determining whether or not they are experiencing some underlying pathology. While this approach can be used to rank z-scores across modalities, the magnitude of the z-score can also be informative, to provide insight into how much of an outlier this individual is in that modality (which may or may not indicate the presence of a pathology).

For the Bayesian optimisation to work efficiently (i.e., faster than exhaustive search across modalities), it needs to take advantage of covariance across modalities in individual differences. In Figure 4A, the order that the opmisation procedure tests the modalities (Movie, resting-state fMRI, task BOLD, T2, FA, GM) reflects this covariance structure. This provides prior information that the optimisation algorithm can combine with some random initial samples (numbers 1–3 in Figure 4A, left) to build a Gaussian process regression model to predict the modality with minimum z-score (in this case number 4, T2).

By chance, the proportion of participants for whom the algorithm finds the modality with the minimum z-score is 0.67 (given that it sampled four modalities in total). When the Bayesian optimisation algorithm utilises the estimated covariance structure from the training dataset, the proportion increases to >0.72 on average (results in Figure 4B are presented from 100 replications). We see that if the modality ordering is chosen randomly (rather than based on covariance across individuals from the training set) the average proportion of participants where the minimum modality is selected approximates that expected by chance (i.e., 0.67). We also see that this translates into an increase in the estimated minimum z-score found when using the optimisation algorithm compared to the random modality ordering. This difference between true and random ordering of modality search space is relatively modest (approximately 5%). However, the dataset used in this example has very few modalities and thus a restricted search space and has a relatively limited sample size. Also, we used somewhat coarse pre-processing and summary statistics. Applying this approach to on-going data collection in much larger projects or at clinical neuroimaging centres that scan large numbers of people, alongside the myriad of different MRI scan modalities available, means that this approach could be substantially improved and used much more powerfully for biomarker discovery.

## Discussion

Here, we have outlined the Active Acquisition approach for optimising multimodal neuroimaging scan protocols. The analytical examples are intended to illustrate the potential utility of Active Acquisition; by using this approach important decisions about the scan do not need to be in advance; how long to scan for, what modalities to acquire, which regions of the brain to focus on. Rather, the precise nature of the scanning protocol is determined online, adapting to the individual in the scanner, optimising acquisition for a given set of circumstances. Our current goal has been to outline several broad scenarios that suggest how Active Acquisition could progress and its general potential, rather than provide evidence of a specific biomarker or indeed specific pipelines or analysis approaches. Here, we discuss future potential directions for Active Acquisition, in particular for diagnosis and stratification as well as for biomarker discovery. We envisage these two directions developing along independent but complementary lines. We also consider some practical issues that need to be overcome to take the approach forward and maximise its potential for clinical and scientific neuroimaging.

### Clinical diagnosis

Perhaps the more obvious use case for Active Acquisition is in clinical diagnostics, and the stratification of individuals into subgroups. Incorporating Active Acquisition could lead to either shorter scanning sessions, or more accurate and more reliable data collection. Multiple imaging modalities are typically collected in a diagnostic clinical scanning session, many of which end up being unnecessary for accurate diagnosis. If the scanning session can be terminated early, when sufficient diagnostic certainty has been reached (as in Scenario 1), there would be a significant reduction in scanning time, reducing patient discomfort and scanning costs. Equally, by optimising the order of the scans (as in Scenario 2), tailored to the targeted disorder, this would potentially remove the need to collect all modalities, leading to the same benefits in terms of time, cost and patient comfort.

Alternatively, Active Acquisition could be used to produce more accurate diagnoses and to optimise certain modalities for clinical use that are currently not used in clinical settings. Active acquisition could make use of scanning time and resources more efficient; collecting repetitions of important scans (until a sufficient signal-to-noise ratio has been reached), or changing the scanning resolution or field-of-view to focus on potential abnormalities. This may be of particular use in relatively low signal-to-noise imaging modalities. For example, the pattern of brain damage presented Scenario 1 (focal ischaemic stroke) is evident even on very low resolution and low signal-to-noise structural scans; however, other neurological conditions may have far more subtle abnormalities and other modalities (e.g., arterial spin labelling, diffusion tensor imaging, resting state or task BOLD scans) have lower signal-to-noise, and may benefit from more spatially focused, repeated data acquisition.

A pertinent issue facing neuroimaging research in clinical samples is how to deal with heterogeneity within patient groups; particularly common in chronic neuropsychiatric diseases. The “average” best scanning protocol sequence may well not be optimal at identifying clinically relevant abnormalities in a specific individual. Potentially, different scans may be optimal for a given diagnosis in different individuals and at different points in the natural history of a disease. One major strength of active acquisition approaches is that they can more easily locate an individual patient’s “sweet-spot” from a large menu of possible scan types/parameters in a time-efficient manner, without having to exhaustively search through all possibilities.

### Biomarker discovery

Finding biomarkers that sensitively detect individual variability linked to clinical and scientific questions is an important precursor to improving diagnosis and stratification. The application of active acquisition illustrated in Scenario 3 presents a radically different way to achieve this: actively searching for modalities or scanning parameters give abnormal readouts for a single individual. This approach contrasts with the current typical approach to biomarker discovery which can be characterised as choosing a set of modalities prior to scanning that are thought to be related to the clinical question, and then assessing them on a large group of patients and controls or subgroups of patients, to provide sufficient statistical power to detect average group differences. Active acquisition also has the benefit of attempting to focus on modalities only when they are likely to be abnormal for an individual relative to a normative dataset, which is potentially much more powerful than the comparison of group averages, as well as leading intuitively to clinical applications of personalised medicine. Active acquisition also has the advantage of relying less on relatively arbitrary decisions that lead to a limited number of modalities being acquired, which means that the clinically-relevant sweet-spot for data acquisition is more likely to be found.

Active Acquisition could also avoid the potential problem of scanning protocols being determined based on biased or inaccurate previous studies. Given the replication ‘crisis’ in biomedical research, such issues are becoming increasingly recognised as a serious problem in medical imaging. Active optimisation approaches (such as in Scenario 3) involve repeatedly cycling between prediction and hypothesis testing on out-of-sample data, and as such are less susceptible to data overfitting. Equally, active optimisation approaches like these also involve a form of implicit “pre-registration” ( Lorenz *et al*., 2017). This makes it harder to engage in certain questionable research practices (e.g., p-hacking, post-hoc hypothesising ( Poldrack *et al*., 2017)) that are currently thought to hamper the development of neuroimaging biomarkers.

One additional advantage of active optimisation is that it is able to estimate how an individual varies from normality across the whole of the search space, despite only sampling a subset of the modalities tested included in the space. While the gains observed were relatively minor in the current example, where only six modalities organised along one searchable dimension were considered, the potential benefit would grow as the space becomes larger and multidimensional. Using the optimisation algorithm to map out the entire possible space offers the potential for a very rich, but efficiently collected, description of how an individual differs from normality. The search space mapped out could involve observing multiple optima in a given individual and estimating modalities with higher and lower than typical signal. Subsequent offline higher-level modelling (e.g., clustering or other data reduction approaches) could then be applied across individuals to find frequent patterns of abnormality from across all modalities.

### Need for different types of normative datasets

One major limiting step to the development of active acquisition is the need to have well-characterised variability across individuals in both healthy or ‘normal’ participants as well as clinical samples and relevant subgroups. Achieving this will require developing large and representative datasets from which to derive estimates of between-individual covariance. Currently for Scenario 1, the normative dataset is simply the n=7 healthy controls; in reality the size of normative cohorts will have to be much larger.

Some simpler applications of Active Acquisition could be built with existing normative datasets. For example, when the problem involves deciding when to stop collecting more data because a sufficient signal-to-noise ratio has been reached, increasing confidence in the inferences made from these data. Other approaches could take advantages of new acquisition methods such as the very rapid multi-contrast images at the start of a scan ( Skare *et al*., 2018) or synthetic imaging which are then used to decide whether to collect slower, higher resolution scans. To utilise these types of scans, existing datasets could be utilised to create sufficiently large normative models.

However, for other applications, such as when searching across modalities (Scenarios 2 and 3), the benefits of Active Acquisition may be most evident when the space of possible modalities/parameters to be considered is large but structured in some way. Indeed, while at present only a small number of imaging modalities are employed clinically, more modalities could be useful but only for stratifying specific subgroups. An accurate understanding of the covariance between modalities/scan parameters relevant to the clinical or scientific question will be necessary for maximising the benefit from these approaches

In Scenario 3, where the optimisation algorithm maps out where an individual is maximally abnormal, understanding the covariance across imaging modalities in a healthy control group (possibly controlling for factors such as age) may suffice. Existing large-scale projects to produce large normative databases have focused on small numbers of modalities collected in large numbers of people (e.g., UK Biobank ( Sudlow *et al*., 2015), Human Connectome Project ( Van Essen *et al*., 2013) and the Cam-CAN dataset presented here). One possible approach is to use meta-analyses of different imaging modalities to try to estimate covariance structure across modalities (capitalising on the fact that different large-scale projects have some shared modalities but also differ from each other). An even better approach would be to have large-scale data collection projects that explicitly seek to quantify covariance across many different imaging modalities/scan parameters. Ideally, this would involve many different representative individuals being scanned, but each with different subsets of modalities/scan parameters; subsequently, a large, comprehensive covariance matrix across individuals can be assembled out of the incomplete datasets from each individual. These normative datasets will allow active searching for how individual patients vary from normality across many modalities, useful for biomarker discovery, without requiring dedicated large multimodal datasets for each clinical condition. Approaches such as Bayesian optimisation with Gaussian processes will allow us to start with relatively few assumptions (i.e., only approximate similarity across modalities near each other in the experimental search space which can be based on health control data); importantly, the approach should work for individuals even when there are areas of the experimental space that deviate from the normative data.

There are also likely to be some situations, however, where acquiring targeted multi-modal normative datasets for specific clinical conditions will also be important. For example, when performing diagnostics rather than discovery of biomarkers (more like in Scenario 2). In these situations, bespoke multimodal datasets may be necessary to arrive at a very specific quantification of the covariance between different modalities, in order to accurately guide the sequential decision making. In such situations, particularly, with rare disease groups, acquisition of such datasets would be far more challenging and may not be practical.

At present, it is unknown how much benefit we can derive by collecting additional normative databases covering a wider array of scan types. The potential benefit depends on whether or not types of scans not already collected in existing large normative samples capture clinically or scientifically useful variability and the potential benefit of that clinical and scientific value. Equally, it depends on the scientific or clinical question, and whether we already know the optimal scans for assessing individual variability. To assess the potential benefit, one approach is to start with relatively small-scale normative data collection with many types of scans in the same individuals; analysis of covariance between scans will allow us to estimate individual variability not captured by the small number of modalities typically collected. Consequently, we will be able to estimate how much benefit can be gained by individually-tailoring scan sequences compared to acquiring the same small subset of scans on everybody.

### Methodological considerations

All methodological approaches come with costs and benefits; with Active Acquisition approaches one concern is that early mismeasurement can lead to serious failures later on. For example, in Scenario 1, this could result in terminating scans prematurely without collecting sufficient data; or, e.g., in Scenario 2, this could involve travelling down the wrong branch of the decision tree. In such situations, important information for diagnosis or biomarker discovery may not be collected. This cost of using active approaches will be most acute when the underlying covariation between scan modalities is well understood and the optimal scan type is known. In contrast, the way that we currently collect data in many exploratory studies (e.g., UK Biobank), it is likely that optimal scans for assessing variability in an individual are being omitted. This reflects the classic exploration versus exploitation trade off well-known in computer science. There is a potential risk when designing adaptive experiments that the acquisition function is too exploitative and acquired data will fail to be broad enough to allow for future serendipitous discovery about unrelated scientific or clinical questions. The choice of an appropriate exploratory acquisition function guiding data collection has the potential to balance between efficient imaging while also estimating individual variability across a larger space of different types of scans, that may be relevant to other questions or future studies.

For the benefits of active exploration to be maximised, many choices have to be made regarding the acquisition function to guide exploration, how to decide when to stop searching, how to quantify abnormality or predict an individual’s classification. We have suggested several simple illustrative scenarios, but each comes with its own specific challenges and future directions. There is a long history of methodological developments for adaptive studies in clinical situations ( Cornfield *et al*., 1969) ( Hauskrecht & Fraser, 2000)( Alagoz *et al*., 2010). Future work is needed to incorporate some of the more sophisticated approaches developed in these other domains to neuroimaging and ideally combine them multivariate classification and clustering approaches increasingly commonly used with MRI. In Scenarios 2 and 3, work is needed to understand what happens when there is not a single optimum modality to maximally quantify abnormality (Scenario 3) or multiple equally good paths through the decision tree (Scenario 2).

Future work is also needed to evaluate how to robustly quantify the abnormality of an individual’s scan, considering the large number of voxels and possibly heterogeneous or diffuse pathologies. The examples presented were designed to clearly illustrate different adaptive approaches rather than show state-of-the-art outlier detection or age classification. As such, performance at age classification in Scenario 2 was based on whole-scan based on summary statistics and a transparent decision tree technique; therefore, performance is expected to be considerably inferior to more sophisticated voxelwise approaches. Equally, in Scenario 3, we chose to only update the optimization with a single summary statistic, but multiple, complementary measures from a single scan could be in calculated in parallel and used to update multiple points in the search space simultaneously. More generally, there are a whole range of future potential avenues for developing and applying more sophisticated, but less transparent approaches to be explored in future work.

Another major consideration is the inherent trade-offs between how long the analysis takes set against the potential benefits of the adaptive approach and potential time savings. Current image analysis pipelines are often very slow and potentially costly in terms of computational processing power, making near real-time analysis infeasible. For example, the image analysis in Scenario 1 would be possible in near real time, (full pre-processing and analysis pipeline using a standard, quad-core personal computer in < 1 minute); however, for Scenarios 2 and 3, data was processed by a high-performance compute cluster with some of the pipelines (e.g., fitting tensor models to calculate fractional isotropy or non-linear image registration) being far slower than would be feasible for active acquisition. These timing challenges have to be offset against any potential gains from adaptive approaches. To address these challenges will require the use of parallelization and dedicated computational hardware that could be used to substantially improve speed as well as optimized pipelines, maximising speed; this will bring processing time down to a fraction of the current time (as has been achieved with e.g., fMRI for brain computer interfaces) with minimal loss of image quality. Finally, recent developments in deep learning offer considerable promise; deep learning approaches, that are slow and costly to train, requiring large datasets, but are very fast to apply. Existing work suggests that many of the more time-consuming steps of pre-processing could be accomplished in near real time using these approaches. For example, structural MR image can undergo an analogue of a complete pre-processing pipeline in a matter of seconds ( Cole *et al.*, 2017)., making near real-time applications applied to multimodal imaging practical.

Finally, from an MR physics perspective, there are also a number of limitations and challenges. Actively altering the field-of-view and resolution (as suggested in Scenario 1 where the scan zooms in on the site of injury) for 3D structural imaging may not have any benefits (in terms of time saved, increased resolution) given inherent trade-offs between tissue contrast, signal to noise and number of measurements acquired. However, a similar approach could be taken with other imaging modalities (e.g., arterial spin labelling, diffusion imaging) where increased signal to noise from restricting the number of slices or increasing the resolution may be beneficial. Equally, there may be different sources of information that different resolutions and fields-of-view could acquire (e.g., rapidly assessing geometry at higher resolution and tissue contrasts at a lower resolution).

In summary, here we have presented Active Acquisition, a novel conceptual approach to how neuroimaging data could be collected. We have utilised advances in optimisation algorithms and harnessed large publicly-available neuroimaging databases to develop Active Acquisition. This approach embeds data analysis into the acquisition process, allowing information to be obtained and employed for making online decisions about the optimal scans or parameters for a given clinical or scientific goal. While Active Acquisition is still at the embryonic stage, our intention with this manuscript and the illustrative examples contained herein, is to provide the groundwork for future conceptual and experimental work aimed at optimising the acquisition of neuroimaging data for clinical and scientific purposes.

## Software availability

Underlying code used to perform this method is available from GitHub: https://github.com/ActiveNeuroImaging/MultimodalActiveAcquisition.

Archived source code at time of publication: http://doi.org/10.5281/zenodo.1478784 ( Leech & Cole, 2018)

Licence: MIT

## Data availability

The anonymised and pre-processed data used in all scenarios is available from the same Github repository as the code. The MRI data has been provided in an anonymised format and registered to an average template space.

Github: https://github.com/ActiveNeuroImaging/MultimodalActiveAcquisition

Zenodo. Dataset 1: ActiveNeuroImaging/MultimodalActiveAcquisition: v1.0 http://doi.org/10.5281/zenodo.1478784 ( Leech & Cole, 2018)

This dataset is available under a MIT license.

The original data used in Scenarios 2 and 3 was from the Cambridge Centre for Ageing and Neuroscience (CamCAN) project dataset: https://camcan-archive.mrc-cbu.cam.ac.uk/dataaccess/

## Media

**Video 1: General illustrative video of one active acquisition approach for structural neuroimaging.** (If video fails to play, it is also available from the Github repository).

https://doi.org/10.6084/m9.figshare.7296920.v1 ( Cole *et al*., 2018)
