# Supplementary material for: Active Acquisition for multimodal neuroimaging
Source: Wellcome Open Res. 2019 Sep 23;3:145. Originally published 2018 Nov 14. [Version 2] doi: 10.12688/wellcomeopenres.14918.2 (PMC6807153; doi:10.12688/wellcomeopenres.14918.2)
Supplement: Peer review history

# Peer review: Reviewer response for version 2

**DOI:** 10.21956/wellcomeopenres.16937.r36546

**Published:** 2019-10-21

**Authors:** Ron Kikinis

**Affiliations:** 1 Surgical Planning Laboratory, Harvard Medical School, Boston, MA, USA; 2 University of Bremen, Bremen, Germany

I am satisfied with the changes.

Is the rationale for developing the new method (or application) clearly explained?

Yes

Is the description of the method technically sound?

Partly

Are the conclusions about the method and its performance adequately supported by the findings presented in the article?

Partly

If any results are presented, are all the source data underlying the results available to ensure full reproducibility?

Partly

Are sufficient details provided to allow replication of the method development and its use by others?

Partly

Reviewer Expertise:

Medical image computing.

I confirm that I have read this submission and believe that I have an appropriate level of expertise to confirm that it is of an acceptable scientific standard.

# Peer review: Reviewer response for version 1

**DOI:** 10.21956/wellcomeopenres.16265.r34521

**Published:** 2019-03-14

**Authors:** Ron Kikinis

**Affiliations:** 1 Surgical Planning Laboratory, Harvard Medical School, Boston, MA, USA; 2 University of Bremen, Bremen, Germany

This is a paper about a method called active acquisition. The authors propose a framework where neuroimaging sequence selection is iteratively optimized while the patient is on the table using an active learning approach. The method is currently a simulation. At this point the paper is an outline of a research strategy not a presentation of a method that is actually working.

 In practical terms, the speed of the computation will be critical. If the computation increases the time that the patient spends in the scanner, this will result in resistance to using this approach.

 My main concern is that the three scenarios are spread widely and mostly talk about the concept. In my opinion the authors should limit themselves to one scenario and flesh it out with enough detail that the work could be duplicated.

**Introduction**:

- “Active learning also has another important feature; they involve a prediction and testing cycle..” they or it?
- “using information gained from previous scans actively seek out brain abnormalities or make diagnostic predictions…” should read “to actively seek..”

**Methods**:

 Scenario 1: detection of stroke pathology

 Choice of parameters: 

- How about a short summary of Dataset 1? The paper should be self-contained.
- “At each iteration the scan…” which scan? Is it at full resolution or, as implied in the introduction, at reduced resolution? How much reduction?
- What is a normative sample and where is it coming from?

Outlier distance:

- When you say median distance, what do you mean? Distance in voxels, signal intensities, something else?

Scenario 2: choose a modality

- Again, provide a short description of the data set, so the reader does not have to visit a different webpage just to know what data you are using. Also, which modalities are you planning to use in the simulation and why? I went to the Cam-CAN webpage and modalities include task based fMRI and MEG. It is not clear to me how such acquisitions could be performed dynamically at need.

Scenario 3: discovery of individual differences.

- “we attempt to actively learn which modality an individual is most likely to be an outlier in” - Please reword.
- The section data acquisition provides the details that would have been helpful to have when reading the scenarios. There were no cross-references in the previous sections. The scenario descriptions 2 and 3 do not mention that only a subset of the Cam-CAN data is being used.

**Results**:

 Scenario 1: Figure 2 is not clear to me. What do you show when you mean composite?

 Scenario 2:  It would be helpful to have two examples: a young and an old person and how their trajectory in the regression is different.

 Scenario 3: I do not understand what the results are. “Here we simulated closed-loop Bayesian optimisation used to discover the modality for a given individual (from the holdout dataset) where the negative outlier distance is most (i.e., relative to normative data from the training dataset), shown in Figure 4.”

**Discussion**:

- The authors should begin the discussion of the results in the three scenarios and how they relate to existing literature. Just stating that the current goal was to outline several broad scenarios before going on to a broad discussion of what the simulated technique could be used for is not to my liking and rather unconventional.

Is the rationale for developing the new method (or application) clearly explained?

Yes

Is the description of the method technically sound?

Partly

Are the conclusions about the method and its performance adequately supported by the findings presented in the article?

Partly

If any results are presented, are all the source data underlying the results available to ensure full reproducibility?

Partly

Are sufficient details provided to allow replication of the method development and its use by others?

Partly

Reviewer Expertise:

Medical image computing

I confirm that I have read this submission and believe that I have an appropriate level of expertise to state that I do not consider it to be of an acceptable scientific standard, for reasons outlined above.

# Peer review: Reviewer response for version 1

**DOI:** 10.21956/wellcomeopenres.16265.r34533

**Published:** 2019-01-23

**Authors:** Diego Vidaurre

**Affiliations:** 1 Department of Psychiatry, Oxford Centre for Human Brain Activity (OHBA), University of Oxford, Oxford, UK

In this paper, Cole *et al*. advocate for using adaptive decision procedures during the data acquisition stage such that data collection is optimised with regard to the question at hand.

 I think this is conceptually a quite valuable contribution. Although the concepts are clear, my only  negative comment is that it is sometimes slightly hard to follow in the details – although this does not get in the middle of the general message too much.

 For example: "To do this, we converted each modality to a z-score, then performed a factor analysis (using Matlab) and calculated a single factor". One eventually gets an idea of what the authors mean, but this could be expressed more clearly.

 - What do they mean by convert each modality to a z-score, exactly? How do they do it? First I understood that they normalised each voxel across subjects within each modality, but that's not what it's meant.

 - Perform factor analysis; is it done across subjects? i.e. the input factor is (no. subjects by modalities)? To walk the reader through these details and being more explicit would make the reading  more amenable.

 Another example: why the objective in Scenario 3 is to find the minimum z-score? Could the authors elaborate to make it more accessible?

 Another slightly negative comment is that perhaps Example 1 is somewhat trivial. Isn't it standard practice to do this? In any case, I guess it works to illustrate the point.

 I particularly liked the Discussion, which I found informative and honest. I am a bit worried about the normative data sets, given that differences in acquisition and preprocessing can make a huge difference (for example, HCP and UKBiobank data are hardly comparable between them).

 Relatedly, would it be possible to extend this paradigm of "choosing modality" to "tuning the preprocessing pipeline"? Admittedly the acquisition is the most costly thing, but the same idea could guide perhaps questions like whether to work in volumetric or surface space, which we know makes a big difference in the HCP for example.

 In this line, I was wondering whether having different versions of each modality (for example sensor and source space in MEG) would enrich and improve the optimisation in the end? Intuitively I would think that should be the case.

 Minor:

 "the objective underlying function is unknown and costly to evaluate: -> I would say that either it's unknown or is costly. One can't evaluate (no matter at what cost) something that is unknown?

Is the rationale for developing the new method (or application) clearly explained?

Yes

Is the description of the method technically sound?

Yes

Are the conclusions about the method and its performance adequately supported by the findings presented in the article?

Yes

If any results are presented, are all the source data underlying the results available to ensure full reproducibility?

Yes

Are sufficient details provided to allow replication of the method development and its use by others?

Yes

Reviewer Expertise:

Neuroscience, Machine Learning

I confirm that I have read this submission and believe that I have an appropriate level of expertise to confirm that it is of an acceptable scientific standard.

# Peer review: Reviewer response for version 1

**DOI:** 10.21956/wellcomeopenres.16265.r34522

**Published:** 2019-01-21

**Authors:** Hugo G Schnack

**Affiliations:** 1 Department of Psychiatry, Brain Center Rudolf Magnus, University Medical Center Utrecht, Utrecht University, Utrecht, The Netherlands

This manuscript discusses the possible need to change the way neuroimaging is used for diagnostic purposes. The authors argue that, currently, an (MRI) examination uses a fixed set of scan acquisitions (a multimodal imaging protocol), i.e., the same protocol for every patient. This possibly means that either some scans are acquired that are unnecessary for diagnosing this patient, and/or some other scans that are not part of the protocol should have provided better diagnostic information regarding this patient. The authors suggest that changing to an adaptive way of acquiring neuroimaging data -which they call 'active acquisition'- could address this issue of obtaining more efficiently better neuroimaging data, both for clinical and scientific use.

 Their method basically starts with acquiring some base scan, which is then analyzed. The information obtained from this first scan is then used to decide what will be the next scan, and so on. The method thus uses a decision tree to determine the multimodal acquisition set for each individual patient. Each patient's acquisition set can thus differ both in length (number of scans) and in kind (modality, resolution, ...) from other patients' sets.

 The authors illustrate their ideas with three examples of possible implementations. The authors also state that there are quite a number of (challenging) issues that need to be addressed before such an approach could be of use. In the following I will discuss some of these points and raise some other issues that were not addressed (enough) in the current manuscript.

Time

 The factor time (or cost) is one of the main ingredients in the proposed method. Although the authors state that faster processing tools need to be developed for their approach, the reader may at least expect an educated estimate of the time that can be saved, by not acquiring unnecessary scans. This gain in time is compensated by a loss of time due to image processing and analysis. Image reconstruction can take quite some time, images probably have to be transferred to a dedicated computer system for further processing, images need to be processed and analyzed and decisions need to be converted to setting up the next acquisition. The current manuscript does not mention at all times, neither actual times for the steps taken in their examples nor estimates for the steps not currently executed. This information, together with estimates of savings due to new developments, should provide the reader a good impression of whether or not the proposed approach is viable.

Economics

 While time saving definitely benefits the patients, the effect on cost is less clear. If scanning takes less time this might reduce the cost of scanning, but it is not clear how this is compensated by increased costs for compute servers, data servers, network equipment and computer programmers on the one hand, and the (for some scenarios) required building of normative databases on the other hand.

 Also, although current examination protocols may (to some extent) lead to acquisition of scans that are unnecessary for the diagnosis of this patient, these scans may later turn out to be useful, either for additional diagnostic purposes, for contributing to (new) normative databases, or for scientific research. It happens quite often that neuroimaging data acquired for some (scientific) purpose can be used to answer new and different research questions. Being able to use these data lowers the cost involved in these new studies. It would be worthwhile to have some discussion about this, including estimates regarding the economical feasibility of the proposed method.

Validity

 While walking along different investigation paths for different individual cases (scenario 1) clearly is a sensible approach for making clinical diagnosis, for quantitative research this is different. Scenario 2 is used to show how the proposed method can be used 'to quantify [an individual's] relationship to a normative sample'. In this example, each patient' age is predicted based on 1 out of 6 different selections of scans (Figure 3). It is unclear what the effect of individual-based feature selections is on the resulting output (age, in this case). One may expect bias here and differences in accuracy and reliability, all because of using scans that differ in number and/or modality. Apart from showing that the overall prediction accuracy is comparable to that of a state-of-the-art traditional modeling approach (as has been done), the authors should provide a thorough analysis, comparing the different performance measures in a detailed manner. More generally, the potential effects of the proposed method on the statistical validity should be discussed.

 Example scenarios - some specific comments:

Scenario 1.

 This is a very straightforward example perfectly illustrating a potential application.

 Some more information, however, may be expected regarding the following potential issues.

- I can imagine that the process described here is already part of the current examination process, in the sense that an MRI technician decides, after a first quick scan, how the acquire the next (high-resolution) scan. How does the time-saving (if at all) relate to the potential loss in accuracy?

This scenario uses 'the highest outlier distance':

- What if the lesion (or brain abnormality) is too small to have an effect that is statistically detectable?
- What if the lesion lies half-way two thick slices from the first scan?
- What if there are two (or more) lesions in different slices, or if there is longer lesions oriented orthogonal to the slice orientation?

It is not clear what is depicted in Figure 1's right panel. Please clarify the caption.

Scenario 2.

 This example is not very convincing, for two reasons:

 1. The age estimation model is based on summary statistics rather than fully exploiting the richness of the multimodal datasets.

 2. The resulting age prediction is categorical, while a fully continuous prediction may be expected (especially because of the first author's expertise in this field).

Scenarios 2 and 3.

 The authors, at the end, state that more advanced ways to incorporate the methods are needed. I recommend that the authors carry out such an implementation themselves, for at least one of these scenarios (preferably no. 2). Since, as the authors acknowledge, the advantages from the current implementations are modest (scenario 3), such an implementation would be helpful to fully show the advances of their proposed method.

 All in all, the current manuscript reports about a very interesting approach to innovate the way neuroimaging data are acquired. However, to convince the reader of the potential of this approach, more supportive data and discussion is needed.

Is the rationale for developing the new method (or application) clearly explained?

Yes

Is the description of the method technically sound?

Yes

Are the conclusions about the method and its performance adequately supported by the findings presented in the article?

Partly

If any results are presented, are all the source data underlying the results available to ensure full reproducibility?

Partly

Are sufficient details provided to allow replication of the method development and its use by others?

Yes

Reviewer Expertise:

Neuroimaging, data analysis, machine learning

I confirm that I have read this submission and believe that I have an appropriate level of expertise to confirm that it is of an acceptable scientific standard, however I have significant reservations, as outlined above.
